# Supplementary material for: Transcriptomic Signature and Pro-Osteoclastic Secreted Factors of Abnormal Bone-Marrow Stromal Cells in Fibrous Dysplasia
Source: Cells. 2024 Apr 30;13(9):774. doi: 10.3390/cells13090774 (PMC11083355; doi:10.3390/cells13090774)
Supplement: Supplementary file 1 [file cells-13-00774-s001.zip › Supplementary figures and legends.pdf]

Figure S1

Schematic representation of the study design and workflow. **A)** Briefly, bone marrow stromal cells were cultured and isolated from a mouse model with doxycycline-inducible expression of $G\alpha_s^{R201C}$ in cells of the osteogenic lineage in the appendicular skeleton through passage enrichment and negative immunoselection against hematopoietic markers CD45 and CD11b. After that, cells from each mouse were cultured in two groups. At 80% confluency, cultures were transferred to FBS-depleted media and $G\alpha_s^{R201C}$ expression was induced in one of the groups by adding 5 mg/mL of doxycycline. **B)** Human BMSCs were cultured from surgical waste FD tissue and bone marrow specimens from healthy volunteers. At 80% confluency, cells were transferred to FBS depleted media. Plasma was obtained from FD patients. **C)** Media was collected from human and mouse cells 48h later, concentrated by filtration, and used to determine cAMP and secreted factor concentrations. Attached cells were lysed with TRIzol for RNAseq. FD patient plasma was used for detecting BMSC-secreted factors in circulation.

Figure S2

Pathways affecting several biological processes are enriched by genes found within the FD Signature. Using a statistical threshold of adjusted $p<0.01$, genes within the signature were loaded into the STRING database to determine potential protein-protein interactions of differentially regulated genes in FD. The most highly enriched processes involved “Extracellular matrix organization” (Cluster 1, C1), “Cellular metabolism” (C2), “Mesenchymal cell proliferation” (C3), “ATPase activity” (C4), “cAMP signaling” (C5), and “Metallopeptidase activity” (C6).

Figure S3

Cultured BMSCs derived from healthy volunteers (HVs) and patients with FD released pro-inflammatory cytokines, but differences were unable to be detected. Additionally, several factors were undetectable (IL-10, IL-12, IL-13, IL-17, G-CSF, and GM-CSF).

Figure S4

Additional cytokines expressed by cultured murine BMSCs. No differences were detected. IL-4, IL-9 were undetectable.

Fig S1

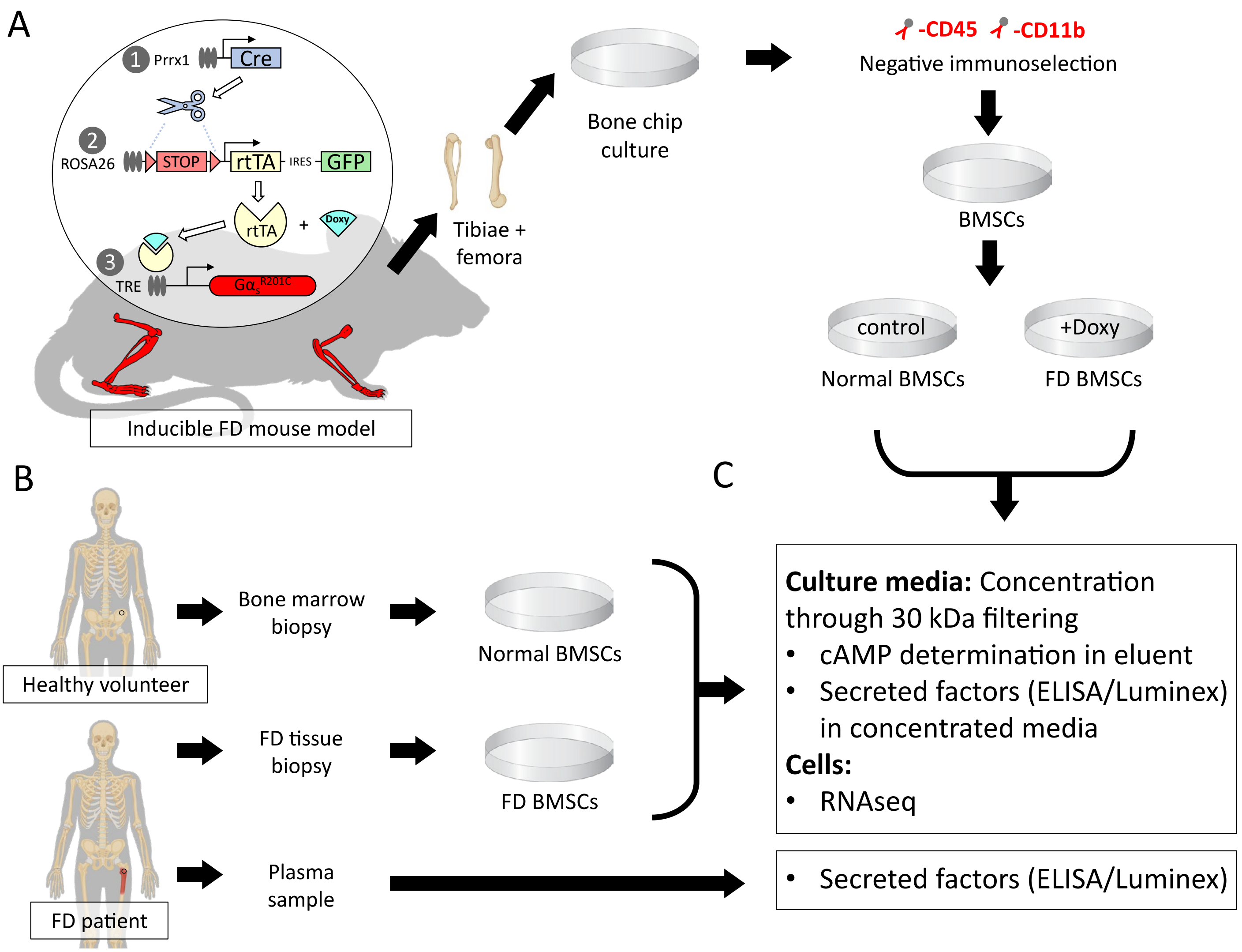
A
1 Prrx1
Cre
2
ROSA26
STOP
rtTA
IRES
GFP
rtTA
+
Doxy
3
TRE
$G\alpha_s^{R201C}$
Inducible FD mouse model
Tibiae +
femora
Bone chip
culture
-CD45 -CD11b
Negative immunoselection
BMSCs
control
+Doxy
Normal BMSCs
FD BMSCs
B
Healthy volunteer
Bone marrow
biopsy
Normal BMSCs
FD patient
FD tissue
biopsy
FD BMSCs
Plasma
sample
C
Culture media: Concentration through 30 kDa filtering
• cAMP determination in eluent
• Secreted factors (ELISA/Luminex) in concentrated media
Cells:
• RNAseq
• Secreted factors (ELISA/Luminex)

Fig S2

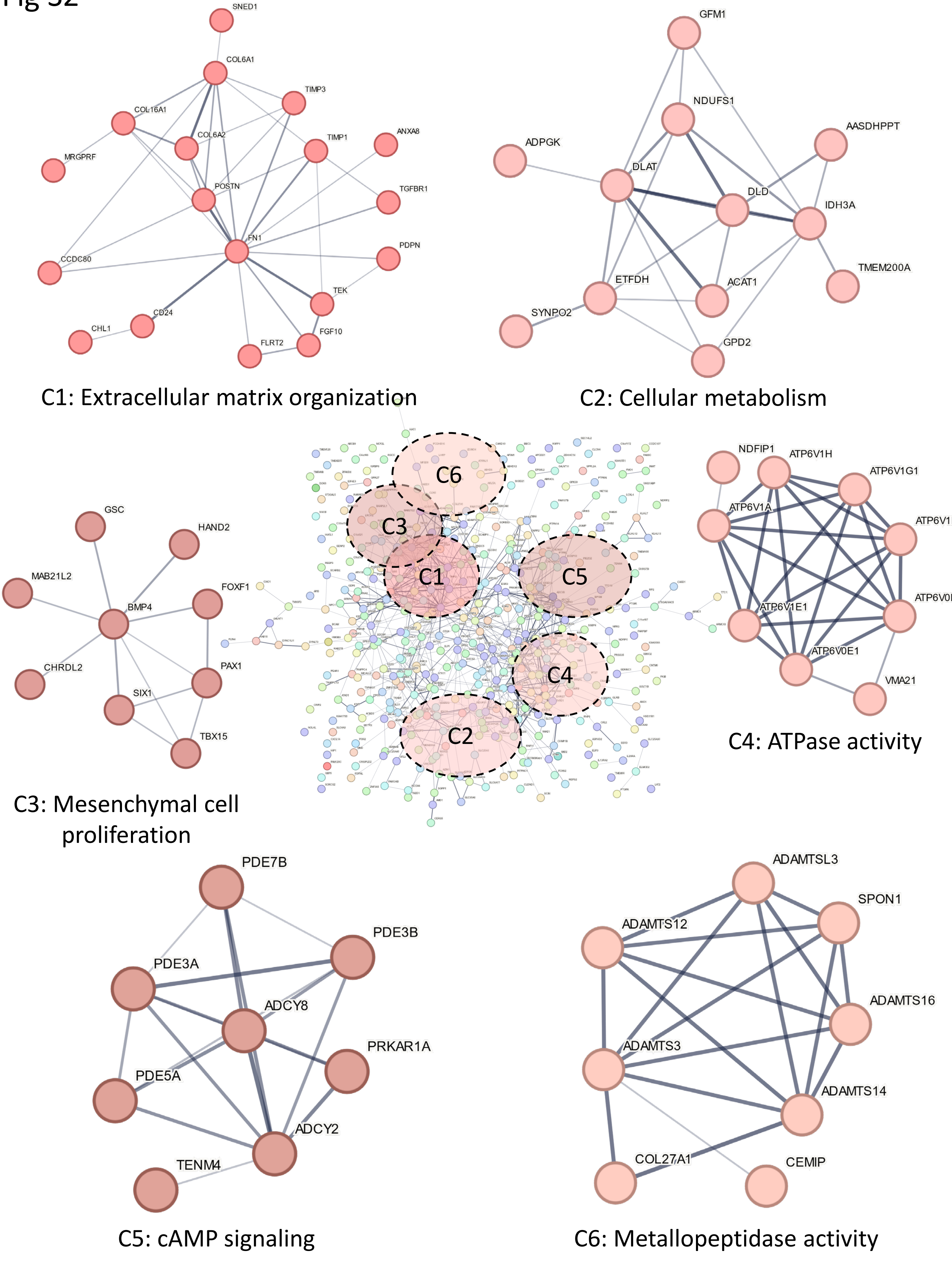

SNED1
COL6A1
TIMP3
COL16A1
COL6A2
TIMP1
ANXA8
MRGPRF
POSTN
TGFBR1
FN1
CCDC80
PDPN
TEK
CD24
CHL1
FGF10
FLRT2
C1: Extracellular matrix organization
GFM1
NDUFS1
AASDHPPT
ADPGK
DLAT
DLD
IDH3A
TMEM200A
ETFDH
ACAT1
SYNPO2
GPD2
C2: Cellular metabolism
C6
C3
C1
C5
C4
C2
GSC
HAND2
MAB21L2
FOXF1
BMP4
CHRDL2
PAX1
SIX1
TBX15
C3: Mesenchymal cell proliferation
NDFIP1
ATP6V1H
ATP6V1G1
ATP6V1A
ATP6V1D
ATP6V0D1
ATP6V1E1
ATP6V0E1
VMA21
C4: ATPase activity
PDE7B
PDE3B
PDE3A
ADCY8
PRKAR1A
PDE5A
ADCY2
TENM4
C5: cAMP signaling
ADAMTSL3
SPON1
ADAMTS12
ADAMTS16
ADAMTS3
ADAMTS14
COL27A1
CEMIP
C6: Metallopeptidase activity

Fig S3

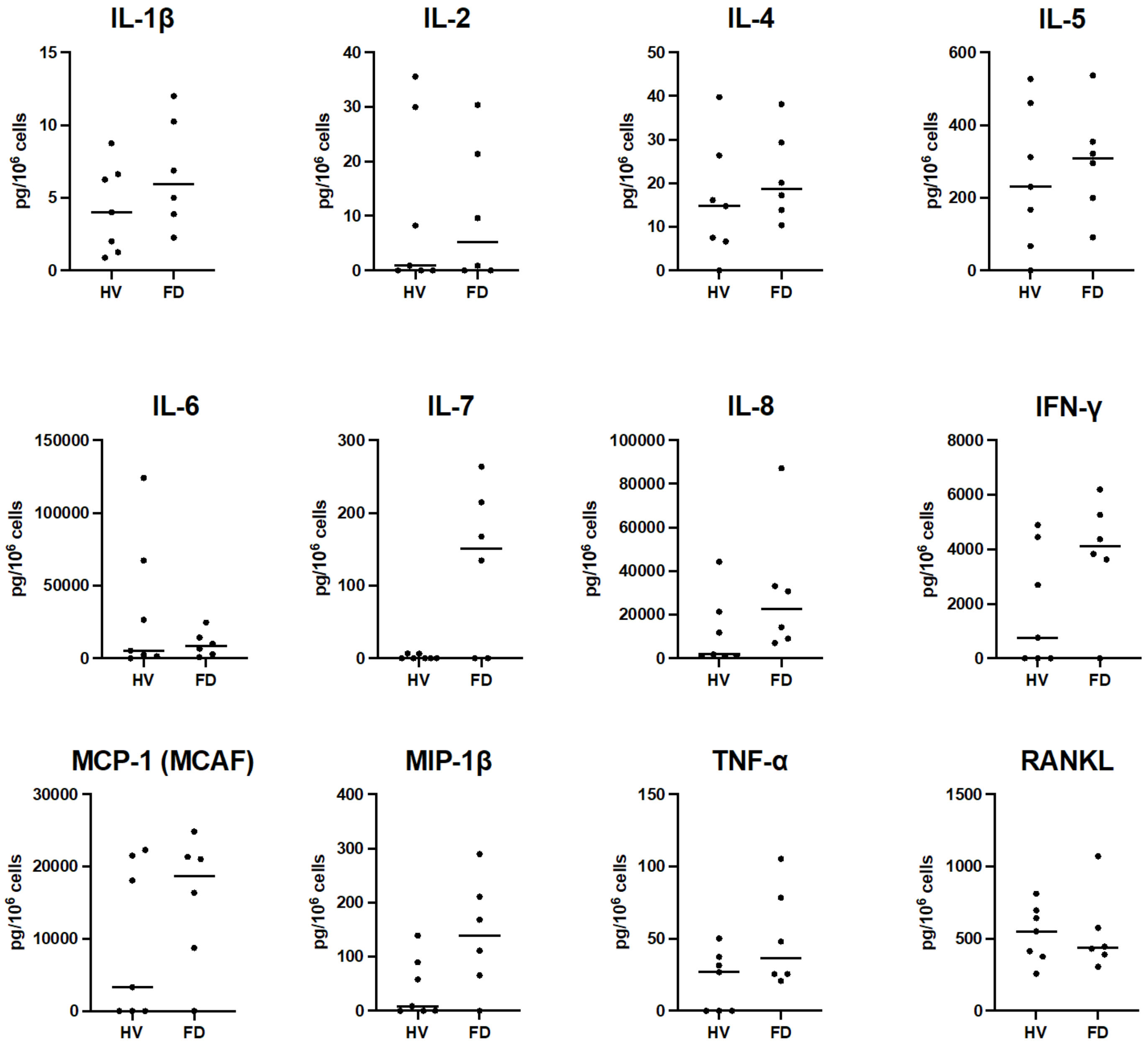
IL-1β
IL-2
IL-4
IL-5
IL-6
IL-7
IL-8
IFN-γ
MCP-1 (MCAF)
MIP-1β
TNF-α
RANKL
pg/10^6 cells
HV
FD

Fig S4

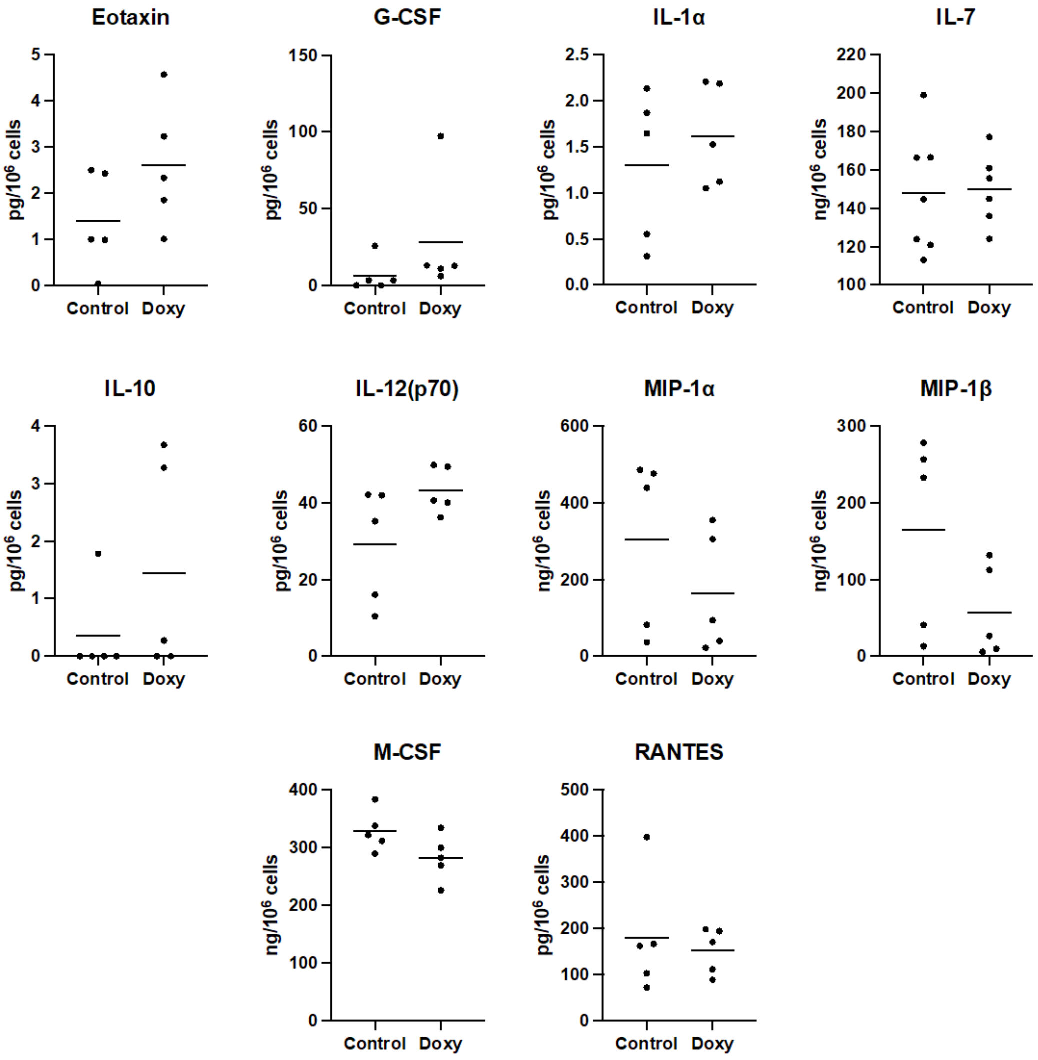
Eotaxin
G-CSF
IL-1α
IL-7
IL-10
IL-12(p70)
MIP-1α
MIP-1β
M-CSF
RANTES
pg/10⁶ cells
ng/10⁶ cells
Control
Doxy
