# Supplementary material for: Transcriptomic Signature and Pro-Osteoclastic Secreted Factors of Abnormal Bone-Marrow Stromal Cells in Fibrous Dysplasia
Source: Cells. 2024 Apr 30;13(9):774. doi: 10.3390/cells13090774 (PMC11083355; doi:10.3390/cells13090774)
Supplement: Supplementary file 1 [file cells-13-00774-s001.zip › Tables S1-S2 - Patient and Donor Demographics.pdf]

Table S1. FD patient plasma donor demographics

**Characteristics (N=57)**	**N (%) or Mean (range)**
Females	32 (56%)
Age at blood draw (years)	22 (4-58)
Skeletal burden score	35.46 (0.5-75)
MAS features	
Skin hyperpigmentation	42 (74%)
Precocious puberty	26 (46%)
Growth hormone excess	23 (40%)
Hyperthyroidism	20 (35%)
Hypophosphatemia	23 (40%)
Cushing syndrome	4 (7%)

Table S2. BMSC donor demographics

**Characteristics**	**FD patients N (%) or Mean (range)**	**Healthy volunteers N (%) or Mean (range)**
Females	3 (50%)	1 (16.66%)
Age at sample donation (years)	13.33 (3-32)	27.66 (22-36)
Culture heterozygosity (% GNAS p.R201C/H mRNA)	17.16% (7-55%)	N/A
Sample source location		
Iliac crest		6 (100%)
Femur	6 (100%)	
